# Laparoscopic versus open surgery for perihilar cholangiocarcinoma: a multicenter propensity score analysis of short- term outcomes

**DOI:** 10.1186/s12885-023-10783-9

**Published:** 2023-05-03

**Authors:** Min Wang, Tingting Qin, Hang Zhang, Jingdong Li, Xiaxing Deng, Yuhua Zhang, Wenxing Zhao, Ying Fan, Dewei Li, Xuemin Chen, Yechen Feng, Siwei Zhu, Zhongqiang Xing, Guangsheng Yu, Jian Xu, Junjie Xie, Changwei Dou, Hongqin Ma, Gangshan Liu, Yue Shao, Weibo Chen, Jun Liu, Jianhua Liu, Xinmin Yin, Renyi Qin

**Affiliations:** 1 Department of Biliary–Pancreatic Surgery, Affiliated Tongji Hospital, Tongji Medical College, Huazhong University of Science and Technology, Wuhan, 430030 Hubei China; 2 Department of Hepatobiliary Surgery, Affiliated Hospital of North Sichuan Medical College, Hepatobiliary, Pancreatic and Intestinal Diseases Research Institute of North Sichuan Medical College, Nanchong, 637000 China; 3 Department of Ruijin Hospital, Shanghai Jiao Tong University School of Medicine, Shanghai, 310000 China; 4 Department of Hepatobiliary, Pancreatic and Minimal Invasive Surgery, Zhejiang Provincial People’s Hospital, People’s Hospital of Hangzhou Medical College, Hangzhou, 310003 China; 5 Department of General Surgery, the Affiliated Hospital of Xuzhou Medical University Xuzhou, Jiangsu, 221000 China; 6 Department of the Second General Surgery, Sheng Jing Hospital of China Medical University, Liaoning, 110000 China; 7 Department of Hepatobiliary Surgery, the First Affiliated Hospital of Chongqing Medical University, Chongqing, 400016 China; 8 Department of Hepatopancreatobiliary Surgery, Third Affiliated Hospital of Soochow University, Suzhou, 213003 China; 9 Department of Hepatobiliary Surgery, Hunan Provincial People’s Hospital, The First Affiliated Hospital of Hunan Normal University, Changsha, 410005 Hunan China; 10 Department of Hepato-Pancreato-Biliary Surgery, the Second Hospital of Hebei Medical University, Shijiazhuang, 050017 Hebei China; 11 Shandong Provincial Institute of Dermatology and Venereology, Shandong Academy of Medical Sciences, 27397 Jingshi Road, Jinan, 250022 China

**Keywords:** Perihilar cholangiocarcinoma, Laparoscopic, Open resection, Short-term outcomes

## Abstract

**Background:**

Laparoscopic surgery (LS) has been increasingly applied in perihilar cholangiocarcinoma (pCCA). In this study, we intend to compare the short-term outcomes of LS versus open operation (OP) for pCCA in a multicentric practice in China.

**Methods:**

This real-world analysis included 645 pCCA patients receiving LS and OP at 11 participating centers in China between January 2013 and January 2019. A comparative analysis was performed before and after propensity score matching (PSM) in LS and OP groups, and within Bismuth subgroups. Univariate and multivariate models were performed to identify significant prognostic factors of adverse surgical outcomes and postoperative length of stay (LOS).

**Results:**

Among 645 pCCAs, 256 received LS and 389 received OP. Reduced hepaticojejunostomy (30.89% *vs* 51.40%, *P* = 0.006), biliary plasty requirement (19.51% *vs* 40.16%, *P* = 0.001), shorter LOS (mean 14.32 *vs* 17.95 d, *P* < 0.001), and lower severe complication (CD ≥ III) (12.11% *vs.* 22.88%, *P* = 0.006) were observed in the LS group compared with the OP group. Major postoperative complications such as hemorrhage, biliary fistula, abdominal abscess, and hepatic insufficiency were similar between LS and OP (*P* > 0.05 for all). After PSM, the short-term outcomes of two surgical methods were similar, except for shorter LOS in LS compared with OP (mean 15.19 *vs* 18.48 d, *P* = 0.0007). A series subgroup analysis demonstrated that LS was safe and had advantages in shorting LOS.

**Conclusion:**

Although the complex surgical procedures, LS generally seems to be safe and feasible for experienced surgeons.

**Trial registration:**

NCT05402618 (date of first registration: 02/06/2022).

**Supplementary Information:**

The online version contains supplementary material available at 10.1186/s12885-023-10783-9.

## Background

Perihilar cholangiocarcinoma (pCCA) is a devastating disease with an annual incidence of one to two cases per 100,000 individuals [1]. Radical surgery is the most important potentially curative treatment for pCCA. Meanwhile, radical surgery is also undoubtedly one of the most difficult and sophisticated skills for surgeons [2]. Recent advances in surgical techniques and perioperative management have offered increased resectability and improvement in surgical outcome; however, post-operative morbidities and mortality remain a problem [3].

Laparoscopic surgery (LS) has been increasingly used in all types of hepato-pancreato-biliary resections including pancreatectomy and hepatectomy [4–6]. In liver surgery, LS presented improved postoperative outcomes compared with open approach [7]. Recently, there has been increasing enthusiasm for performing laparoscopic pancreaticoduodenectomy (PD), which has been demonstrated to be feasible and safe, with outcomes that are comparable to open PD [8]. The surgical procedures for pCCA entail perihilar biliary tract resection with extended hepatectomy, which requires concomitant vascular resection and reconstruction or even combined hepatopancreatoduodenectomy. A major challenge is the complex anatomical location of the lesion, which is in close proximity to the portal vein (PV), hepatic arteries, and liver parenchyma [9, 10]. Thus, there are few reports on laparoscopic resection for pCCA due to the complexity of the surgical procedure. Till now, LS resection for pCCA was considered to be still in its infancy [11]. However, the available evidence on LS for pCCA is limited; LS cannot be considered as a dogmatic contraindication to pCCA. It thus is imperative to undertake large-scale multicenter analyses to investigate in the technical feasibility and safety of LS for pCCA [12].

Herein, we compared perioperative outcomes of LS and open operation (OP) in 654 pCCA patients in China before and after a propensity score-based analysis. To our knowledge, this is the largest series of LS compared with OP for pCCA to be investigated to date.

## Methods

### Patients and data collection

**Fig. 1:**
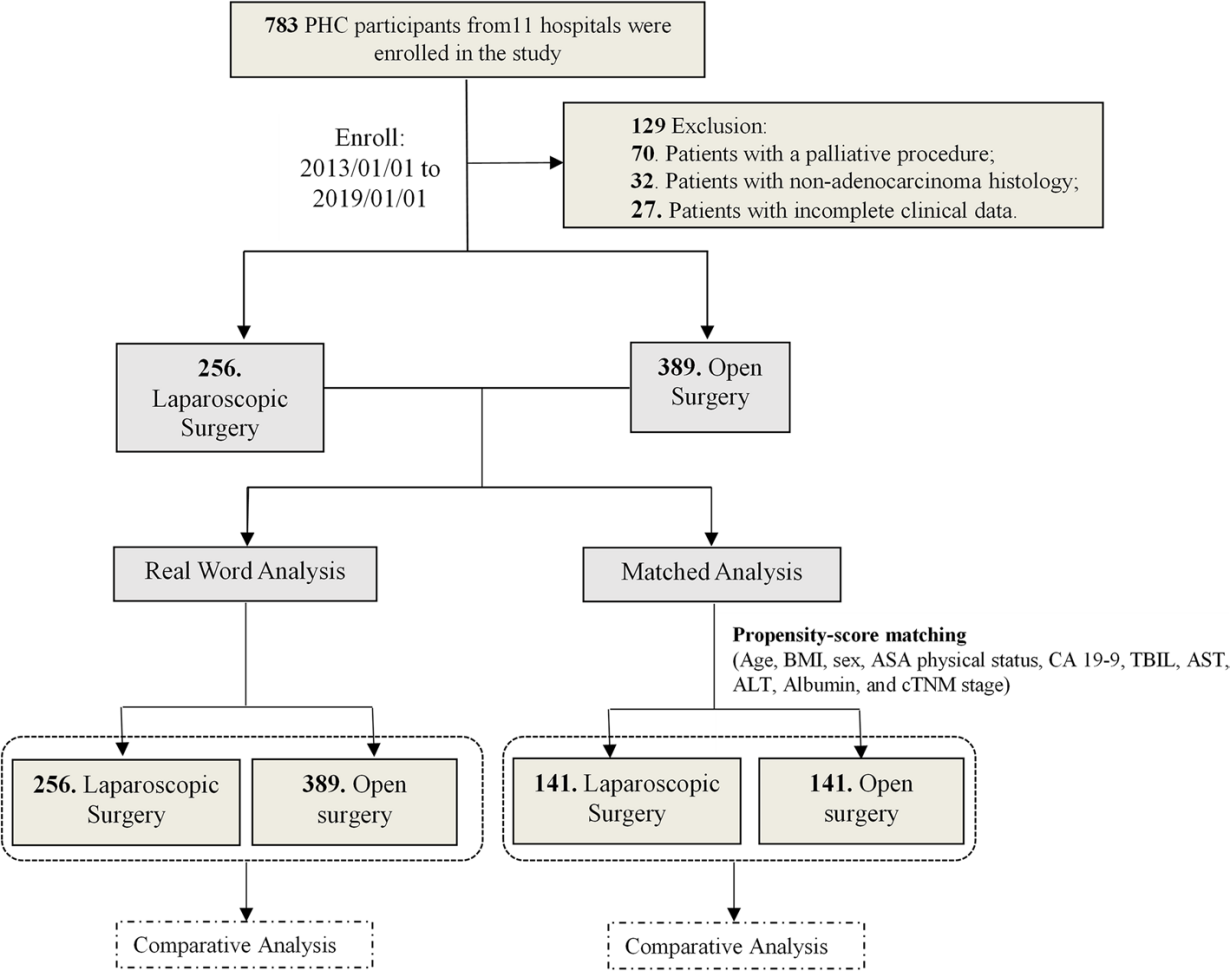
Study flow

A retrospective review of institutional databases from 11 hospitals in China identified 645 pCCA patients who underwent radical surgery between January 2013 and January 2019, including 256 patients underwent LS and 389 underwent OP. An intention-to-treat design was used, such that cases converted to OP were included in the LS cohort. All cases were histologically confirmed pCCA. The exclusion criteria were as follows: (1) patients with peritoneal seeding or metastasis to the liver, para-aortic lymph nodes, or distant sites; (2) patients with non-adenocarcinoma histology; and (3) patients with incomplete clinical data. The detailed study flow was shown in Fig. 1 and Supplementary Table 1. Ethical approval was given for the study by the ethics committee/Institutional Review Board of Ethics Committee of Tongji Hospital (approval number: TJ-IRB20220531). The need for informed consent was waived by the ethics committee/Institutional Review Board of Ethics Committee of Tongji Hospital (approval number: TJ-IRB20220531) because of the retrospective nature of the study. This research was also registered at ClinicalTrials.gov (identifier: NCT05402618; date of first registration: 02/06/2022). All the work followed the ‘Sex and Gender Equity in Research-SAGER-guidelines’ and has been reported in line with the STROCSS criteria [13].

### Operative technique

LS was defined as total laparoscopic surgery (TLS), where both resection and digestive reconstruction were completed laparoscopically. The hepatectomy with en bloc resection of the caudate lobe and extrahepatic bile duct and regional lymph node dissection was applied in both laparoscopic and open surgery. Extended resections (left/right trisectionectomy), arterial vascular encasement, vascular resection/reconstruction, biliary stent in place were needed for patients based on Bismuth stage.

### Variables and definitions

Data collection included demographic, clinical and oncologic data. Patients were staged according to the National Comprehensive Cancer Network (NCCN) Guideline Version 2.2019 Edition tumor node metastasis (TNM) clinical staging system for pCCA [14]. The local extension of the disease was expressed mainly according to the Bismuth–Corlette classification [15]. Operation time was defined as the time from skin incision or trocar placement to complete skin closure. Intraoperative blood loss (IBL) was carefully recorded by the anesthetist using a vacuum system. Vessel reconstruction was defined as any repair or replacement of major vessels during surgery. Main short-term outcome in this study was postoperative length of stay (LOS), which was defined as the time from surgery to normal discharge. Patients who stayed longer than 30 days or less than three days will be considered as censored. Other short-term evaluations including morbidity and mortality, defined as any complication or death, respectively, which occurred during hospitalization or within 90 days of discharge. Hospital reoperation within 30 days was recorded and postoperative morbidity was evaluated according to the Clavien-Dindo (CD) classification system [16]. Major postoperative complications such as bile leakage, postoperative hemorrhage, and liver failure were classified as previously reported [17–19]. Surgical failure was defined as severe complication (CD ≥ III), or received reoperation during hospitalization, or dead within 30 days after surgery.

### Statistical analysis

Patients that underwent LS were matched in a 1:1 ratio to patients that underwent OP based on the propensity score model [20], with age, BMI, sex, ASA physical status, CA 19–9, preoperative Tbil, AST, ALT, albumin, Bismuth-Corlett type, hepatectomy, operative time, digestive reconstruction, IBL, vascular resection and reconstruction, positive margin, number of lymph nodes, and TMN stage being selected as the covariates. The nearest neighbor matching without replacement was performed with caliper width setting at 0.2.

Continuous data are presented as means with standard deviations (SDs) or medians with interquartile ranges (IQRs) and compared with independent-samples *t*-test or Mann–Whitney U test. Categorical data are presented as numbers and percentages and were compared using the Chi-square or Fisher’s exact test, as appropriate. Logistic regression analysis was used to access the risk factors of adverse postoperative complications (CD stage ≥ III). Cox regression model were used to assess the risk factors associated with postoperative LOS, with the proportional hazard assumption being tested by weighted residual score method. Furthermore, considering the postoperative complication would prolong the LOS during hospitalization, the Fine-Gray model were used to explore the prognostic factors of length of stay, with the adverse postoperative complications and death within 30 days being the competing risk events. Continuous variables will be converted into two or multiple category dummy variables according to the normal range during the regression analysis. All the statistical procedures were conducted using SAS software version 9.40 (SAS Institute, Inc., Cary, NC). Two-sided hypothesis testing with a predetermined level of *P* < 0.05 was considered statistically significant.

## Results

### Participants and baseline characteristics

Baseline characteristics of demographics and preoperative comorbidity of the included 645 resections of histologically confirmed pCCA are listed in Table 1. In raw cohort, most baseline characteristics were comparable in LS and OP group, except for more female patients (49.61% vs. 40.36%; *P* = 0.021), better hepatic function index (lower TBIL, AST, and ALT levels), and lower TNM stage being observed in LS group than in OP group. To overcome the bias from retrospective study, the propensity score matched analyses was conducted, in which 141 pCCA patients undergoing LS were matched with 141 patients undergoing OP. All baseline characteristics were balanced after PSM.

**Table 1:** Baseline characteristics before and after propensity score matching

Demographics	Raw Cohort (*N* = 645)			PSM Matched Cohort (*N* = 282)			
	**LS (*n* = 256)**	**OP (*n* = 389)**	***P*-value**	**LS (N = 141)**	**OP (N = 141)**	***P*-value**	**MSD**
Sex, No.(%)						1.000	< 0.01
Male	129(50.39)	232(59.64)	0.021	86(60.99)	86(60.99)		
Female	127(49.61)	157(40.36)		55(39.01)	55(39.01)		
Age (yrs), mean(SD)	62.83(9.70)	62.10(9.17)	0.333	62.55(9.15)	63.09(9.03)	0.624	0.06
BMI (Kg/cm^2^), mean(SD)	23.12(3.09)	22.75(2.80)	0.112	22.92(3.09)	22.66(2.81)	0.459	0.09
ASA score, No. (%)			0.872			0.471	0.05
I	57(22.27)	82(21.08)		39(27.66)	34(24.11)		
II	156(60.94)	245(62.98)		77(54.61)	87(61.70)		
III	43(16.80)	62(15.94)		25(17.73)	20(14.18)		
CA199 (U/mL), median(IQR)	204.40(69.50 ~ 814.70)	256.00(98.50 ~ 455.00)	0.791	202.30(100.10 ~ 545.80)	187.60(72.70 ~ 369.80)	0.843	0.02
Tbil (μmol/L), median(IQR)	47.85(44.10 ~ 53.65)	131.00(73.10 ~ 230.30)	< 0.001	49.70(44.70 ~ 57.30)	60.50(28.90 ~ 108.80)	0.798	0.03
ALP (μmol/L), median(IQR)	362.65(216.40 ~ 451.15)	327.00(225.00 ~ 462.00)	0.168	345.00(210.00 ~ 440.00)	269.00(189.30 ~ 424.80)	0.856	0.02
AST (μmol/L), median(IQR)	43.00(33.20 ~ 87.60)	89.00(54.00 ~ 156.30)	< 0.001	60.60(35.50 ~ 132.00)	74.00(46.00 ~ 119.30)	0.909	0.01
ALT (μmol/L), median(IQR)	59.10(30.90 ~ 133.65)	115.30(60.00 ~ 183.90)	< 0.001	78.50(36.60 ~ 154.60)	92.90(56.70 ~ 143.40)	0.874	0.02
Albumin (μmol/L), median(IQR)	35.60(33.35 ~ 38.45)	35.90(33.40 ~ 39.20)	0.639	35.60(33.00 ~ 38.20)	35.60(32.50 ~ 39.60)	0.928	0.01
Number of lymphnodes, median(IQR)	12.00(8.00 ~ 16.00)	14.00(5.00 ~ 21.00)	0.760	12.00(6.00 ~ 18.00)	13.00(7.00 ~ 17.00)	0.701	0.05
Maximun Tumor Size(mm), mean(SD)	3.03(1.35)	2.89(1.26)	0.167	2.73(1.18)	2.83(1.29)	0.492	0.08
Preoperative biliary drainage, No. (%)	193(75.39)	268(69.25)	0.091	105(74.47)	90(64.29)	0.064	0.19
Bismuth-Corlett Type, No. (%)			0.895			0.95	0.01
I	72(28.13)	108(27.76)		44(31.21)	39(27.66)		
II	63(24.61)	85(21.85)		24(17.02)	23(16.31)		
IIIa	24(9.38)	35(9.00)		9(6.38)	10(7.09)		
IIIb	33(12.89)	52(13.37)		23(16.31)	27(19.15)		
IV	64(25.00)	109(28.02)		41(29.08)	42(29.79)		
TNM stage, No. (%)			< 0.001			0.725	0.11
Miss	8(3.13)	9(2.31)		4(2.84)	7(4.96)		
I(T1N0M0)	25(9.77)	66(16.97)		22(15.60)	23(16.31)		
II(T2a/2bN0M0)	108(42.19)	148(38.05)		60(42.55)	56(39.72)		
IIIA(T3N0M0)	38(14.84)	31(7.97)		8(5.67)	12(8.51)		
IIIB(T4N0M0)	30(11.72)	25(6.43)		7(4.96)	11(7.80)		
IVA(T, N2M0)	34(13.28)	80(20.57)		30(21.28)	23(16.31)		
IVB(T,N,M1)	13(5.08)	30(7.71)		10(7.09)	9(6.38)		

*PSM* Propensity score matching, *LS* Laparoscopic surgery, *OP* Open operation, *BMI* Body mass index, *Tbil* Total bilirubin, *ALP* A Lkaline Phosphatase, *AST* Aspartic acid aminotransferase, *ALT* alanine aminotransferase

### Perioperative outcomes

Before PSM, 19 patients (7.42%) converted to laparotomy. The mean operative time of the LS group was similar with OP group (353.4 *vs.* 342.1 min,* P* = 0.176). More patients received hepatectomy in LS compared with OP (63.67% vs 55.53%, *P* = 0.013), especially more left hemihepatectomy in LS (47.66% vs 36.76%). Fewer hepaticojejunostomy (39.06% vs 61.44%, *P* < 0.0001), biliary plasty (28.91% vs 48.84%, *P* < 0.001), vascular resection (8.59% vs 23.39%,* P* < 0.0001) and fewer hepaticojejunostomy (43.48% vs 65.48%, *P* < 0.0001) were performed in the LS group compared with OP group. R0 rate, IBL, transfusion rate and volume, as well as caudate lobectomy were similar between groups (Table 2).

**Table 2:** Intraoperative outcomes before and after propensity score matching

Demographics	Raw Cohort (*N* = 645)			PSM Matched Cohort (*N* = 282)			
	**LS(*N* = 256)**	**OP(*N* = 389)**	***P*-value**	**LS (*N* = 141)**	**OP (*N* = 141)**	***P*-value**	**MSD**
Conversion to laparotomy, No. (%)	19(7.42)	0(0.00)	< 0.001^*^	11(7.80)	0(0.00)	< 0.001^*^	4.86
Hepatectomy, No. (%) 0.001^*^						0.076^*^	0.38
Bile duct only	93(36.33)	173(44.47)		66(46.81)	57(40.43)		
Left hemi hepatectomy	122(47.66)	143(36.76)		47(33.33)	52(36.88)		
Right hemihepatectomy	19(7.42)	57(14.65)		12(8.51)	25(17.73)		
Left Segmentectomy	4(1.56)	3(0.77)		2(1.42)	1(0.71)		
Right Segmentectomy	6(2.34)	2(0.51)		5(3.55)	1(0.71)		
Bile duct and part of hepatectomy	12(4.69)	11(2.83)		9(6.38)	5(3.55)		
Operative time (min), mean(SD)	353.39(113.71)	342.10(96.52)	0.176	359.45(105.47)	342.43(92.42)	0.151	0.09
No. lymph node, mean(SD)	12(4.06)	10(3.24)	0.213	12(5.12)	11(4.31)	0.542	0.08
Vascular resection, No. (%)			< 0.001^*^			< 0.001^*^	0.53
None	234(91.41)	298(76.61)		126(89.36)	110(78.01)		
Hepatic artery	13(5.08)	23(5.91)		11(7.80)	8(5.67)		
Portal vein	8(3.13)	14(3.60)		3(2.13)	3(2.13)		
Hepatic artery & Portal vein	1(0.39)	54(13.88)		1(0.71)	20(14.18)		
Biliary plastic, No. (%)	74(28.91)	190(48.84)	< 0.001	61 (43.3)	74 (52.5)	0.153	0.11
Biliary reconstruction, No. (%)			< 0.001			< 0.001	0.41
Choledochojejunostomy	130(56.52)	126(34.52)		68(52.71)	40(30.77)		
Hepaticojejunostomy	100(43.48)	239(65.48)		61(47.29)	90(69.23)		
Caudate lobectomy, No. (%)	138(54.12)	220(56.70)	0.519	85(60.71)	82(58.57)	0.715	0.05
IBL(ml), median(IQR)	200.00(100.00 ~ 500.00)	300.00(200.00 ~ 500.00)	0.685	300.00(150.00 ~ 500.00)	300.00(150.00 ~ 500.00)	0.573	0.07
Transfusion during surgery, No.(%)	80(31.25)	150(38.56)	0.0579	47(33.33)	52(36.88)	0.533	0.07
Transfusion Volume (ml), median(IQR)	0.00(0.00 ~ 600.00)	0.00(0.00 ~ 750.00)	0.9535	0.00(0.00 ~ 600.00)	0.00(0.00 ~ 600.00)	0.680	0.07
R0, No. (%)	218(85.16)	341(87.66)	0.360	115(81.56)	119(84.40)	0.526	0.08

*PSM* Propensity score matching, *LS* Laparoscopic surgery, *OP* Open operation, *IBL* Intraoperative blood loss

^*^Fisher exact test

There were no significant differences with regards to the common postoperative complications between the two groups, except lower heart failure (0.78% *vs.* 3.60%, *P* = 0.0244) and severe complications in LS group (12.11% *vs.* 22.88%, *P* = 0.0006). Patients in LS group required shorter postoperative drainage tube keep time (PDTK) (median [IQR], 8.00[5.00 ~ 11.00] *vs.* 9.00[6.00 ~ 14.00], *P* < 0.0001), and postoperative LOS (median [IQR], 13.00[10.00 ~ 18.00] *vs.* 15.00[12.00 ~ 23.00], *P* < 0.0001) than OP patients. There was no significant difference on reoperation rate or death within 30 or 90 days. The similar incidence of some most common postoperative complications between two surgical groups were still comparable in matched cohort (Table 3).

**Table 3:** Postoperative outcomes according to different BMI level before and after propensity score matching

Demographics	Raw Cohort (*N* = 645)			PSM Matched Cohort (*N* = 282)		
	**LS (*n* = 256)**	**OP (*n* = 389)**	*P*-value ^*^	**LS (*N* = 141)**	**OP (*N* = 141)**	*P*-value ^*^
Major complications, No. (%)						
Hemorrhage	17(6.64)	19(4.88)	0.342	10(7.09)	5(3.55)	0.185
Biliary fistula	19(7.42)	29(7.46)	0.988	14(9.93)	7(4.96)	0.112
Abdominal abscess	20(7.81)	47(12.08)	0.082	17(12.06)	17(12.06)	> 0.999^*^
Hepatic insufficiency	3(1.17)	9(2.31)	0.294	2(1.42)	4(2.84)	0.684^*^
Gastrointestinal fistula	1(0.39)	4(1.03)	0.653	1(0.71)	4(2.84)	0.371^*^
Incision infection	4(1.56)	16(4.11)	0.068	3(2.13)	8(5.67)	0.124
Pneumonia	16(6.25)	28(7.20)	0.640	12(8.51)	9(6.38)	0.496
Renal failure	3(1.17)	10(2.57)	0.216	3(2.13)	5(3.55)	0.723^*^
Heart failure	2(0.78)	14(3.60)	0.024	2(1.42)	3(2.13)	> 0.99^*^
ARDS	3(1.17)	9(2.31)	0.294	3(2.13)	3(2.13)	> 0.99^*^
CD stage ≥ III, No. (%)	31(12.11)	89(22.88)	0.001	26(18.44)	32(22.70)	0.377
Reoperation, No. (%)	5(1.95)	7(1.80)	0.888	4(2.84)	4(2.84)	> 0.99^*^
Death (30d), No. (%)	8(3.13)	24(6.17)	0.082	7(4.96)	12(8.51)	0.235
Death (90d), No. (%)	12(4.69)	34(8.74)	0.050	9(6.38)	15(10.64)	0.200
Time of off-bed activity(d), median(IQR)	4.00(3.00 ~ 7.00)	5.00(3.00 ~ 7.00)	0.406	4.00(3.00 ~ 7.00)	4.00(3.00 ~ 6.00)	0.649
PDTK(d), median(IQR)	8.00(5.00 ~ 11.00)	9.00(6.00 ~ 14.00)	< 0.001	8.00(5.00 ~ 12.00)	9.00(5.00 ~ 13.00)	0.225
Time of ICU(d), median(IQR)	0.00(0.00 ~ 2.00)	0.00(0.00 ~ 1.00)	0.137	0.00(0.00 ~ 1.00)	0.00(0.00 ~ 1.00)	0.762
LOS (d), median(IQR)	13.00(10.00 ~ 18.00)	15.00(12.00 ~ 21.00)	< 0.001	14.00(11.00 ~ 19.00)	15.00(12.00 ~ 23.00)	0.001

*PSM* Propensity score matching, *LS* Laparoscopic surgery, *OP* Open operation, *ARDS* Acute Respiratory Distress Syndrome, *CD* Clavien-Dindo, *PDTK* Postoperative drainage tube keep time, *LOS* Length of stay

^*^Fisher exact test

### Postoperative outcomes according to Bismuth type

In the present study, 328 patients of pCCA had a low Bismuth type (Bismuth I/II) and 317 had a high Bismuth type (Bismuth III/V), with significantly different surgical characteristics and postoperative short-term outcomes. The median LOS was much shorter in the low Bismuth type (15 day; 95%CI, 14 ~ 16) than in the high Bismuth type (18 day; 95%CI, 17 ~ 20, *P* < 0.0001). Furthermore, shorter LOS and PDTK, as well as less transfusion during surgery were observed in patients who underwent LS compared with those who underwent OP among patients with Bismuth types I-II, with other postoperative complications being comparable between the two groups. Among patients with Bismuth types III-V, those who underwent LS showed comparable or better short-term outcomes than the OP group, such as less total postoperative complications (39(32.23)86(43.88), *P* = 0.0393), shorter length of stay (median days, 14 vs. 17 days), and lower rates of severe complications (10.74% vs. 26.02%), demonstrating guaranteed safety of laparoscopic resection for high Bismuth types of PHC. Besides, a significantly less vascular resection and biliary plasty were observed in the LS group (Supplementary table 2–7).

### Subgroup analysis for surgical failure and LOS

**Fig. 2:**
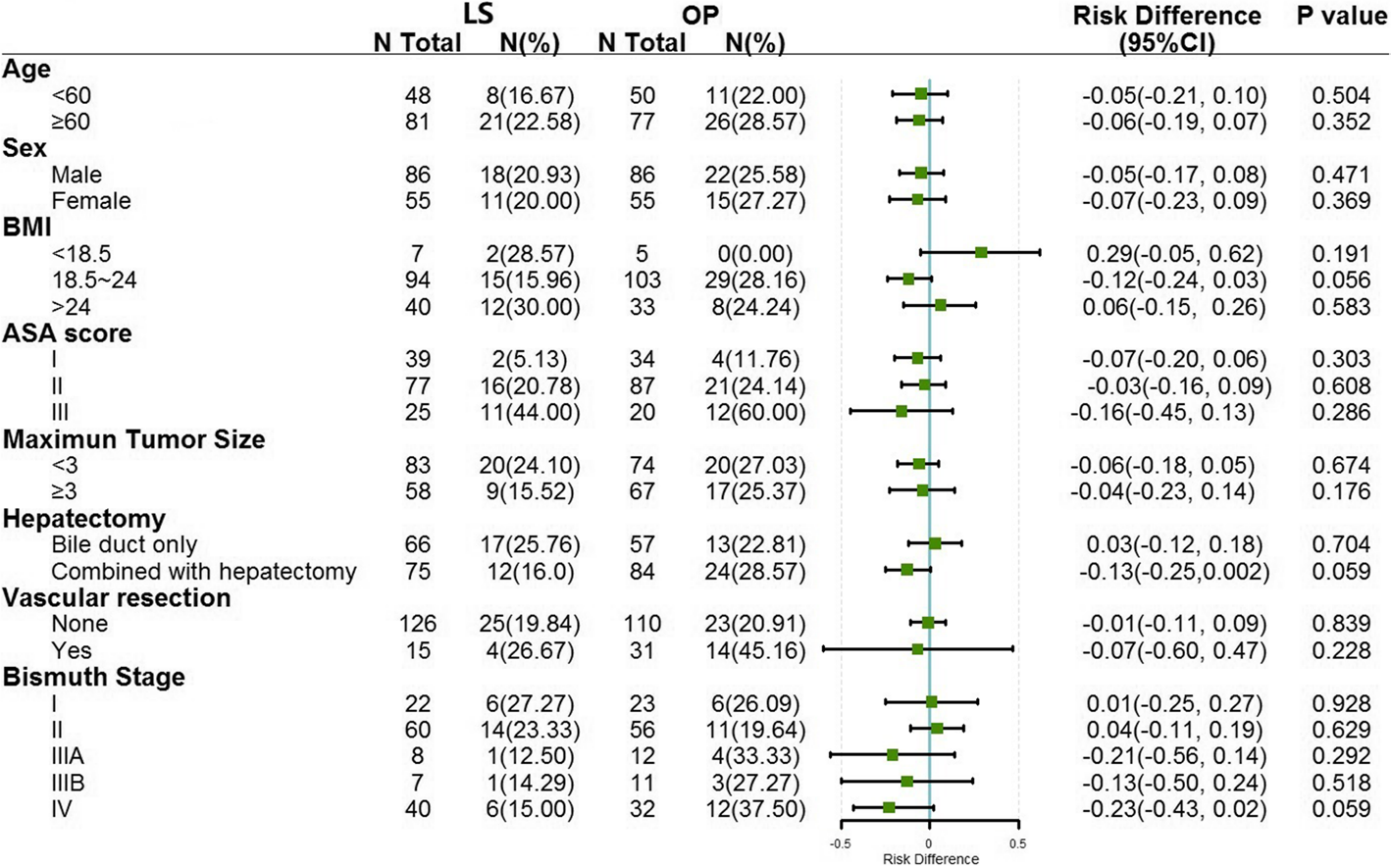
Forest plot of subgroup analysis regarding to surgical failure. Surgical failure was defined as severe complication (CD ≥ III), or received reoperation during hospitalization, or dead within 30 days after surgery.

**Fig. 3:**
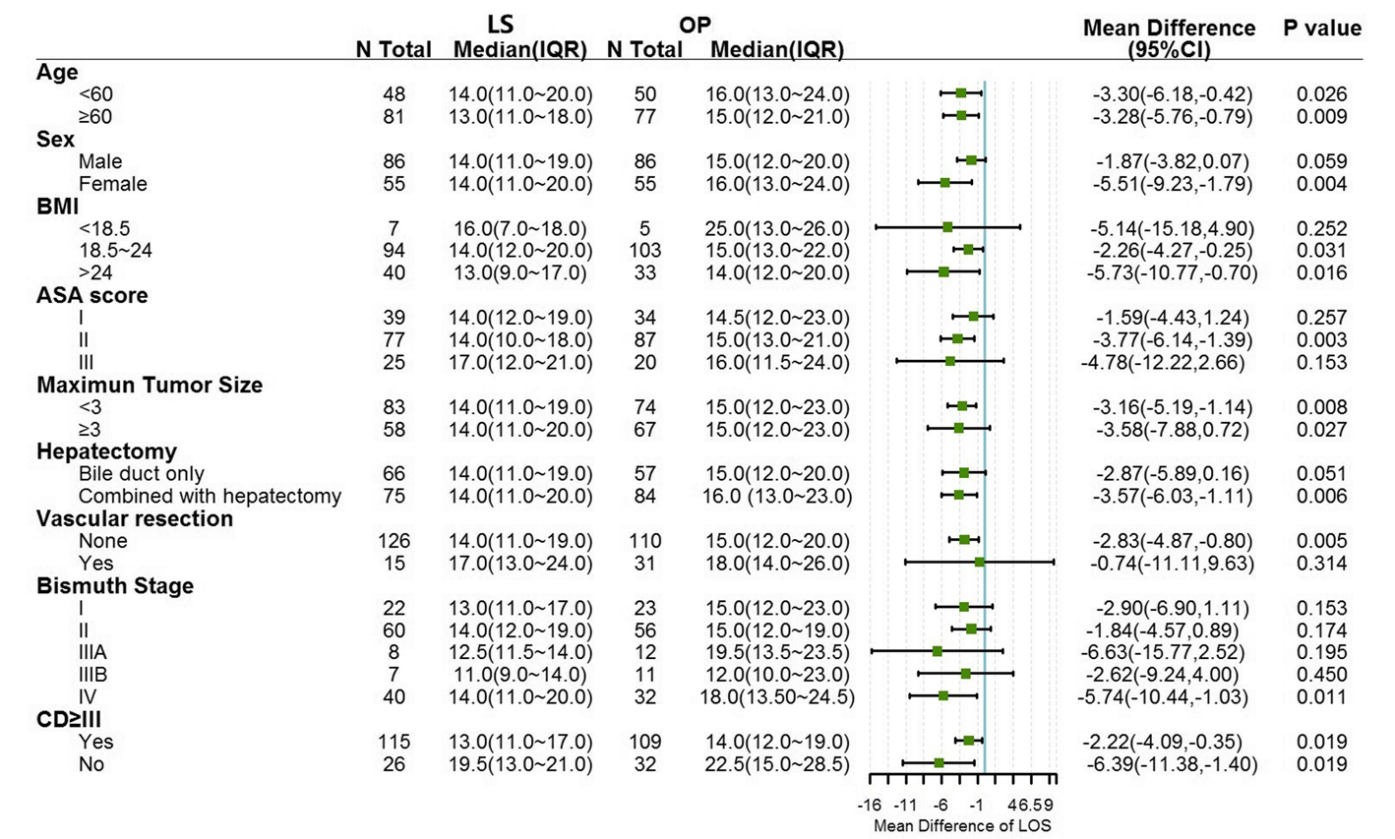
Forest plot of subgroup analysis regarding to length of stay

Subgroup analysis about the surgical failure and LOS was performed according to age, gender, BMI level, ASA score, tumor size, hepatectomy, vascular resection, and Bismuth stage. After PSM, the rate of surgical failure was similar in LS compared to OP group in total (4.30% *vs* 7.20%, *P* = 0.131) and in all subgroups (Fig. 2). However, the subgroup analysis about LOS showed that patients would benefit from LS for rapid recovery, regardless of age, tumor size and severe postoperative complications. Other patients, such as those who were underweight, had ASA score of I or III, or underwent vascular resection, had comparable LOS in LS and OP group (Fig. 3).

### Risk analysis of adverse surgical outcome and length of stay

The risk analysis showed that higher ASA score, more intraoperative blood loss, and more vascular reconstruction were identified independent risk factors of postoperative complications among both matched and unmatched samples (adjusted odds ratio (AOR) > 1, *P* < 0.05 for all) (Table 4). Besides, we found the OP, higher level of CA19-9, as well as some common postoperative complications, and reoperation were associated with longer LOS after adjusting other covariates in matched cohort (Table 5).

**Table 4:** The risk factors analysis of postoperative adverse outcomes according to univariate and multivariate analysis using raw cohort and matched cohort

Risk Factors	Raw Cohort				PSM Matched Cohort			
	**Univariate analysis**		**Multivariate analysis**		**Univariate analysis**		**Multivariate analysis**	
	**HR(95%CI)**	***P* value**	**HR(95%CI)**	***P* value**	**HR(95%CI)**	***P* value**	**HR(95%CI)**	***P* value**
Surgery								
LS	Reference				Reference			
OP	1.68(1.20,2.35)	0.002			0.97(0.60,1.56)	0.904		
Female	0.79(0.57,1.08)	0.141			1.07(0.66,1.75)	0.776		
Age ≥ 60 y	0.95(0.68,1.34)	0.780			0.77(0.47,1.27)	0.305		
BMI, Kg/cm^2^								
18.5	Reference				Reference			
18.5 ~ 24	0.72(0.32,1.64)	0.436			2.09(0.64,6.82)	0.221		
> 24	0.79(0.55,1.13)	0.195			0.98(0.57,1.70)	0.955		
ASA								
I	Reference		Reference		Reference		Reference	
II	1.88(1.22,2.89)	0.004	1.938(1.203,3.121)	0.007	1.75(0.97,3.18)	0.064	2.36(1.23,4.53)	0.010
III	2.41(1.40,4.13)	0.001	2.578(1.388,4.788)	0.003	3.39(1.56,7.38)	0.002	5.11(2.15,12.17)	< 0.001
Tumor size > 3 cm	0.67(0.48,0.95)	0.025			0.88(0.51,1.49)	0.624		
Positive of lymphnodes ≥ 1	1.20(0.85,1.70)	0.295			1.25(0.74,2.10)	0.402		
Lymphnodes > 8	1.56(1.09,2.23)	0.016			1.16(0.68,1.98)	0.584		
Preoperative Tbil > 85.5 μmol/L	1.58(1.146,2.18)	0.005			1.68(0.99,2.85)	0.053		
Preoperative AST > 40 μmol/L	2.20(1.50,3.23)	< .0001			1.97(1.10, 3.54)	0.023		
Preoperative ALT > 40 μmol/L	2.12(1.42,3.18)	0.0003			1.75(0.96,3.20)	0.068		
CA199, U/ml								
≤ 50	Reference				Reference			
50 ~ 400	1.38(0.88,2.19)	0.164			0.98(0.49,1.94)	0.944		
≥ 400	1.45(0.89,2.36)	0.137			1.11(0.51,2.41)	0.788		
Operating time, min								
≤ 200	Reference				Reference			
200 ~ 400	1.35(0.75,2.42)	0.315			2.22(0.90,5.47)	0.082		
≥ 400	2.11(1.13,3.96)	0.020			2.66(1.01,7.01)	0.048		
IBL, ml								
≤ 100	Reference		Reference		Reference		Reference	
100 ~ 500	2.45(1.58,3.80)	< .0001	2.23(1.40,3.55)	< 0.001	2.45(1.27,4.72)	0.008	2.28(1.14,4.54)	0.019
≥ 500	3.41(2.15,5.42)	< .0001	2.75(1.64,4.62)	< 0.001	2.33(1.17,4.62)	0.016	1.54(0.74,3.21)	0.254
Transfusion during surgery	2.00(1.44,2.78)	< .0001			1.43(0.87,2.35)	0.154		
Hepatectomy								
Bile duct only	Reference				Reference			
Left hemihepatectomy	0.80(0.56,1.14	0.212	0.49(0.32,0.74)	< 0.001	1.06(0.617,1.82)	0.835		
Right hemihepatectomy	2.36(1.40,3.96)	0.001	1.02(0.54,1.92)	0.955	1.33(0.633,2.79)	0.453		
Left Segmentectomy	4.29(0.82,22.51)	0.086	4.06(0.66,25.02)	0.131	3.13(0.28,35.41)	0.358		
Right Segmentectomy	1.71(0.42,7.01)	0.453	1.46(0.34,6.22)	0.613	1.56(0.30,8.06)	0.594		
Bile duct and part of hepatectomy	0.91(0.37,2.23)	0.844	1.37(0.47,4.11)	0.576	0.87(0.27,2.75)	0.810		
Conversion to laparotomy	1.21(0.48,3.05)	0.686			1.78(0.53,5.99)	0.349		
Vascular resection								
None	Reference				Reference			
Hepatic artery	1.30(0.65,2.60)	0.461	1.35(0.65,2.82)	0.419	1.00(0.38,2.63)	0.999		
Portal vein	2.95(1.24,7.03)	0.015	1.97(0.71,5.49)	0.196	8.56(0.98,74.49)	0.052		
Hepatic artery & Portal vein	5.97(3.17,11.25)	< .0001	3.47(1.70,7.07)	< 0.001	5.48(1.94,15.48)	0.003		
Vascular reconstruction	3.28(1.44,7.49)	0.005			12.41(1.53,100.65)	0.013	13.18(1.53,113.89)	0.019
Digestive reconstruction								
Choledochojejunostomy	Reference				Reference		Reference	
Hepaticojejunostomy	2.01(1.43,2.82)	< .0001	1.81(1.22,2.70)	0.004	1.80(1.08,2.99)	0.024	2.13(1.21,3.75)	0.009
Biliary plasty	1.33(0.96,1.84)	0.082			0.84(0.52,1.35)	0.459		
TNM stage								
I(T1N0M0)	Reference				Reference			
II(T2a/2bN0M0)	0.78(0.48,1.28)	0.325			0.84(0.42,1.69)	0.617		
IIIA(T3N0M0)	0.84(0.44,1.60)	0.602			1.67(0.58,4.83)	0.342		
IIIB(T4N0M0)	0.62(0.31,1.27)	0.191			1.10(0.36,3.30)	0.872		
IVA(T, N2M0)	1.01(0.58,1.77)	0.960			1.05(0.47,2.34)	0.907		
IVB(T,N,M1)	0.91(0.44,1.91)	0.807			0.80(0.27,2.41)	0.689		

*PSM* Propensity score matching, *LS* Laparoscopic surgery, *OP* Open operation, *HR* Hazard ratio, *BMI* Body mass index, *ALP* A Lkaline Phosphatase, *AST* Aspartic acid aminotransferase, *ALT* Alanine aminotransferase, *IBL* Intraoperative blood loss

**Table 5:** The Cox proportional hazard model analysis of length of stay using raw cohort and matched cohort

Risk Factors	Raw Cohort				PSM Matched Cohort					
	**Univariate analysis**		**Multivariate analysis**		**Univariate analysis**			**Multivariate analysis**		
	**HR(95%CI)**	***P* value**	**HR(95%CI)**	***P* value**	**HR(95%CI)**	***P* value**		**HR(95%CI)**		***P* value**
Surgery										
LS	Reference		Reference		Reference			Reference		
OP	0.67(0.57,0.80)	< .0001	0.74(0.61,0.90)	0.002	0.66(0.51,0.85)	0.001		0.58(0.45,0.75)		< .0001
Female	1.08(0.91,1.27)	0.378			0.83(0.64,1.07)	0.147				
Age ≥ 60y	1.11(0.94,1.32)	0.228			1.10(0.85,1.43)	0.465				
BMI										
18.5	Reference				Reference					
18.5 ~ 24	1.39(0.93,2.06)	0.108			0.84(0.44,1.59)	0.584				
> 24	1.07(0.89,1.29)	0.480			1.14(0.86,1.52)	0.360				
ASA										
I	Reference				Reference					
II	0.91(0.74,1.11)	0.347			0.91(0.68,1.20)	0.494				
III	0.81(0.62,1.06)	0.131			0.73(0.49,1.09)	0.120				
CA199, U/ml										
≤ 50	Reference		Reference		Reference			Reference		
50 ~ 400	0.57(0.46,0.72)	< .0001	0.60(0.47,0.76)	< .0001	0.67(0.48,0.95)	0.025		0.455(0.315,0.658)		< .0001
≥ 400	0.64(0.50,0.81)	0.001	0.59(0.450,0.77)	< .0001	0.75(0.50,1.11)	0.144		0.424(0.279,0.645)		< .0001
Tumor size > 3 cm	1.12(0.94,1.33)	0.214			1.01(0.76,1.33)	0.963				
Lymphnodes > 8	0.91(0.75,1.09)	0.292			1.03(0.78,1.36)	0.855				
Preoperative Tbil > 85.5 μmol/L	0.85(0.73,1.01)	0.057			0.77(0.58,1.02)	0.064				
Preoperative AST > 40 μmol/L	0.69(0.58,0.83)	< .0001			0.83(0.62,1.11)	0.208				
Preoperative ALT > 40 μmol/L	0.59(0.48,0.71)	< .0001	0.74(0.59,0.91)	0.005	0.62(0.46,0.84)		0.002			
CA199, U/ml										
≤ 50	Reference		Reference		Reference				Reference	
50 ~ 400	0.57(0.46,0.72)	< .0001	0.60(0.47,0.76)	< .0001	0.67(0.48,0.95)		0.025		0.46(0.32,0.66)	< .0001
≥ 400	0.64(0.50,0.81)	< .0001	0.59(0.45,0.77)	< .0001	0.75(0.50,1.11)		0.144		0.42(0.28,0.65)	< .0001
Operating time, min										
≤ 200	Reference				Reference					
200 ~ 400	1.13(0.84,1.51)	0.428			1.23(0.79,1.91)		0.353			
≥ 400	0.94(0.68,1.29)	0.683			1.20(0.75,1.93)		0.446			
IBL, ml										
≤ 100	Reference				Reference					
100 ~ 500	0.75(0.62,0.92)	0.004	0.95(0.77,1.17)	0.625	0.90(0.66,1.23)		0.523			
≥ 500	0.49(0.39,0.61)	< .0001	0.54(0.42,0.69)	< .0001	0.66(0.47,0.91)		0.013			
Transfusion during surgery										
Hepatectomy										
Bile duct only	Reference				Reference					
Left hemihepatectomy	1.16(0.97,1.39)	0.100	1.26(1.02,1.55)	0.029	1.02(0.77,1.35)		0.909			
Right hemihepatectomy	0.57(0.43,0.75)	< .0001	0.78(0.57,1.05)	0.104	0.72(0.49,1.07)		0.102			
Left Segmentectomy	0.73(0.32,1.64)	0.444	1.06(0.45,2.47)	0.895	0.81(0.25,2.55)		0.712			
Right Segmentectomy	0.73(0.34,1.54)	0.403	0.88(0.38,2.02)	0.761	0.64(0.26,1.58)		0.336			
Bile duct and part of hepatectomy	0.67(0.41,1.08)	0.098	0.78(0.45,1.37)	0.390	0.86(0.47,1.56)		0.618			
Conversion to laparotomy	0.80(0.50,1.28)	0.351			0.79(0.43,1.44)		0.440			
Vascular resection										
None	Reference				Reference					
Hepatic artery	0.74(0.52,1.06)	0.101			0.79(0.48,1.29)		0.342			
Portal vein	0.71(0.46,1.10)	0.127			0.54(0.24,1.22)		0.138			
Hepatic artery & Portal vein	0.44(0.32,0.61)	< .0001			0.49(0.29,0.81)		0.006			
Vascular reconstruction	0.74(0.50,1.11)	0.141			0.58(0.30,1.12)		0.105			
Digestive reconstruction										
Choledochojejunostomy	Reference				Reference					
Hepaticojejunostomy	0.69(0.58,0.82)	< .0001	0.808(0.662,0.986)	0.035	0.75(0.58,0.98)		0.034			
Biliary plasty	0.93(0.79,1.10)	0.377			1.02(0.80,1.308)		0.868			
TNM										
I(T1N0M0)	Reference		Reference		Reference					
II(T2a/2bN0M0)	0.94(0.73,1.21)	0.636	1.04(0.80,1.36)	0.777	0.97(0.67,1.39)		0.851			
IIIA(T3N0M0)	0.82(0.59,1.14)	0.238	0.95(0.66,1.36)	0.761	0.88(0.511,1.53)		0.655			
IIIB(T4N0M0)	1.43(1.01,2.03)	0.047	2.40(1.62,3.55)	< .0001	1.29(0.72,2.31)		0.388			
IVA(T, N2M0)	0.80(0.60,1.06)	0.119	1.13(0.83,1.54)	0.444	0.82(0.53,1.24)		0.343			
IVB(T,N,M1)	0.95(0.65,1.38)	0.788	1.09(0.74,1.62)	0.665	0.83(0.47,1.48)		0.534			
Abdominal abscess	0.44(0.33,0.59)	< .0001	0.46(0.34,0.62)	< .0001	0.47(0.31,0.70)		0.0002		0.41(0.27,0.63)	< .0001
Hemorrhage	0.44(0.29,0.67)	< .0001	0.39(0.26,0.61)	< .0001	0.50(0.28,0.92)		0.027		0.33(0.18,0.63)	0.0007
Biliary fistula	0.49(0.35,0.68)	< .0001	0.50(0.36,0.71)	< .0001	0.64(0.39,1.04)		0.074		0.54(0.33,0.89)	0.016
Heart failure	0.46(0.25,0.86)	0.016	0.26(0.13,0.51)	< .0001	0.39(0.14,1.05)		0.061		0.18(0.07,0.50)	0.001
Incision infection	0.31(0.17,0.54)	< .0001	0.37(0.21,0.66)	0.001	0.39(0.19,0.80)		0.009			
Pneumonia	0.49(0.35,0.69)	< .0001	0.49(0.34,0.71)	< .0001	0.67(0.41,1.09)		0.105			
ARDS	0.36(0.17,0.75)	0.007			0.45(0.17,1.20)		0.109			
Liver failure	0.37(0.18,0.79)	0.010			0.38(0.12,1.17)		0.092			
Reoperation	0.25(0.10,0.61)	0.002			0.21(0.07,0.65)		0.007		0.308(0.10,0.98)	0.046
Renal failure	0.28(0.12,0.69)	0.005			0.29(0.09,0.90)		0.032			

*PSM* Propensity score matching, *LS* Laparoscopic surgery, *OP* Open operation, *HR* Hazard ratio, *BMI* Body mass index, *ALP* A Lkaline Phosphatase, *AST* Aspartic acid aminotransferase, *ALT* alanine aminotransferase, *IBL* Intraoperative blood loss, *ARDS* Acute Respiratory Distress Syndrome

Furthermore, the Fine Gray model were conducted to further investigate the association between surgical methods and the LOS, with postoperative complications, received reoperation during hospitalization, or dead within 30 days after surgery being identified as competing events when analyzing the LOS. After adjusting competing risks, the open surgery was found to be an independent risk factor of prolonged LOS in both unmatched and matched analysis (Supplemental Table 8).

## Discussion

The short-term outcomes of this retrospective study showed that the LS was favored in LOS and function recovery details such as the duration of postoperative drainage tube keep than OP for pCCA patients. Moreover, the mortality rates, postoperative complication rates, and oncological outcomes were not significantly different between two surgical methods. This is the largest real-world pCCA data to date comparing LS and OP for pCCA. It showed that LS, although challenging, is feasible and safe for pCCA patients. We also performed the PSM to eliminate potential biases and found that the LS group was beneficial for postoperative functional recovery, with no differences in complications and death rate compared to the OP group. The multivariate analysis further confirmed that LS was safe for perioperative complications and could significantly shorten the postoperative LOS.

Although several cumulative evidence with meta-analysis have shown the applicability of LS in pCCA [21–24], the technique is still restricted to a minority of highly experienced surgeons and specialized institutions, and lack of large sample evidence from multicenter. LS has been used for all Bismuth-Corlette types, although it is predominantly used in patients with low stage tumors [25–27]. The Bismuth III-IV accounted for 47.26% in this study, and equal safety and better postoperative recover, reflected by shorter LOS and PDTK, were also found in the LS group compared to OP group in this sample. Furthermore, the feasibility of the laparoscopic approach is attested by a conversion rate (19/256 cases, 7.4% before PSM; and 11/141 cases, 7.8% after PSM), which was much lower than the generally reported surgeries for pCCA [28] and also lower for major hepatectomies [29, 30]. In fact, although the surgical management of pCCA involves most complex technical procedures, such as portal infiltration or arterial encasement, or disease infiltration along the bile ducts, the requirements of an urgent conversion are detected with relative low frequency. Since the surgical field can be easily accessible to operator’s views and thus, some operative procedures with a high degree of technical complexity were more manageable in laparoscopy.

To date, only retrospective studies with small sample size reported the perioperative outcomes of LS and OP regarding the pCCA. The efficacy and safety of LS for pCCA is largely controversial [11, 12, 31, 32]. The only comparative study conducted by Xu et al. reported the robotic resection compared unfavorably to traditional open resection in operative time. No significant difference was found in blood loss, mortality, or length of postoperative hospital stay. While the hospital expenditure was much higher in the robotic group, and the tumor recurrence-free survival was inferior in the robotic group [33]. A study summarized a series of laparoscopic procedures showed there was significantly lower blood loss, fewer intraoperative and postoperative blood transfusions, and shorter LOS in LS group. Both overall and lymphadenectomy-related morbidity were lower in the LS group [34]. In this study, there were no significant differences in operative time, IBL, postoperative complications, or death between the two groups, but shorter LOS in LS group. Besides, the surgical methods, no matter open or laparoscopic, were not associated with adverse postoperative complications in both matched and unmatched samples. Furthermore, the LS showed to be a protective factor of adverse postoperative complication than OP in the univariate analysis. These evidence demonstrated that LS is safe and feasible for pCCA patients.

The most important potential advantage of LS is the increased adoption of postoperative functional recovery pathways and shorten hospital stay [35]. The LS technique can not only allow functional recovery for simple operations, such as LC and herniorrhaphy, but also for complex surgeries, such as LPD, laparoscopic hepatectomy, or laparoscopic gastrectomy [36–39]. Our study observed a protective effect of LS in shortening the LOS with or without considering the postoperative complications being the competing risk events. Perioperative complications would potentially influence the short-term outcome evaluation with life-threatening events, and thus affect the assessment of net cumulative effect of LS in shortening the LOS. Neglecting the existence of these competing risk events will lead inaccurate calculations of the cumulative discharge rate when using the classic survival analysis methods [40, 41]. Until now, few studies considered using the Fine-Gray model to explore the postoperative benefits from LS. PSM was another statistical method to control bias in this study. It has been proposed as a method to overcome selection bias and increase the evidence level in observational and randomized studies [42, 43]. In this study, we demonstrated that fewer hepaticojejunostomy, less vascular resection, shorter LOS, and a trend towards fewer severe complication (CD stage ≥ III) in LS group compared to OP before and after PSM. The results indicated that LS could reliably shorten hospital stays and provide benefits regarding postoperative functional recovery in pCCA patients.

Furthermore, we found that high ASA score, more intraoperative blood loss and hepaticojejunostomy were consistent risk factors of adverse surgical outcome. Besides, more intraoperative blood loss was identified as a risk factor for prolonged LOS in both the common risk model and competing risk model. These results indicated that better preoperative condition and less intraoperative blood loss were associated with better short-term prognosis among pCCA patients. During surgical management of pCCA, most technical complexities are related to the management of portal infiltration, or arterial encasement, or disease infiltration along the bile ducts. Along with these conditions and related risks of vascular injury are generally more manageable in laparoscopy since the surgical field is easily accessible being close to operator’s view and bleedings can be stopped with the use of a temporary clamp if emergency situation occurred. Therefore, the LS would be better to manage intraoperative bleeding, potentially bringing benefits for short-term outcome as recently reported [28, 44]. The preoperative condition of the patient is the most concerned issue in the determination of surgery. Patient selection is a common phenomenon in clinical practice, and it is also a key challenge that still puzzled most surgical experts. In our study, we can find that the rate of vascular resection, postoperative biliary fistula, and liver hepatic insufficiency are very low, suggesting an obvious patient selection prior to surgical management. In daily practice, patients with pCCA will be asked to receive ICG test, liver function testing, and 3D imaging examination to help surgeons calculate the liver volume, assess the liver function, and present exact anatomy of the lesion before surgery. Some surgeons would be relatively conservative to perform radical surgery for pCCA when encountering complex resection and reconstruction, especially for laparoscopic surgery. Despite the difficulty, enthusiasm is growing among surgeons regarding laparoscopic radical perihilar cholangiocarcinoma surgery. The selection of suitable patients for laparoscopic radical perihilar cholangiocarcinoma to help surgeons navigate the learning curve will be the focus of our future research.

To our knowledge, this is the largest case series of pCCA comparing LS and OS techniques. However, some limitations must be noted. First, the retrospective nature of the study is at risk of selection biases and unexpected recall bias cannot be completely ruled out. Second, the multicentric nature of the study is at risk of different selection criteria applied in the participating Institutions. While, due to the complexity of the procedure, there is a paucity of laparoscopic resection available now and this large-sample analysis can provide several important information about the laparoscopic procedures of pCCA. Third, all laparoscopic resections performed by experienced hands were still in the early and exploratory stages and is performed only in select cases. In addition, the centers included in this study were all high-volume referral centers in China. The experience may be difficult to generalize to the surgeons with less intensive training. Nonetheless, given the ongoing debate regarding the complicated procedure, and the current shift toward value-based healthcare reimbursement models, our findings are relevant and timely.

## Conclusions

In summary, this extensive, multi-center study demonstrated that LS does not seem to increase the intraoperative and postoperative risks of pCCA. This report thus serves as a foundation for national protocols aimed at safely implementation of LS in pCCA patients. Larger prospective cohort series and prospective randomized studies in multiple countries are warranted to further investigate this topic.

## Supplementary Information

**Additional file 1.**

## Data Availability

The datasets used and analyzed during the current study are available from the corresponding author on reasonable request.

## Abbreviations

LS: Laparoscopic surgery
pCCA: Perihilar cholangiocarcinoma
OP: Open operation
PSM: Propensity score matching
LOS: Length of stay portal vein
PV: Total laparoscopic surgery
TLS: Tumor node metastasis
TNM: National Comprehensive Cancer Network
NCCN: Laparoscopic pancreaticoduodenectomy
PD: Intraoperative blood loss
IBL: Clavien-Dindo
CD: Interquartile ranges
IQR: Standard deviations
SD: Body mass index
BMI: Total bilirubin
Tbil: A Lkaline Phosphatase
ALP: Aspartic acid aminotransferase
AST: Alanine aminotransferase
ALT: Acute Respiratory
ARDS: Distress Syndrome
PDTK: Postoperative drainage tube keep time

## References

[1] Groot Koerkamp B, Wiggers JK, Allen PJ, Busch OR, D'Angelica MI, DeMatteo RP (2014). American Joint Committee on Cancer staging for resected perihilar cholangiocarcinoma: a comparison of the 6th and 7th editions. HPB (Oxford).

[2] Kimura N, Young AL, Toyoki Y, Wyatt JI, Toogood GJ, Hidalgo E (2017). Radical operation for hilar cholangiocarcinoma in comparable Eastern and Western centers: Outcome analysis and prognostic factors. Surgery.

[3] Tang Z, Yang Y, Zhao Z, Wei K, Meng W, Li X (2018). The clinicopathological factors associated with prognosis of patients with resectable perihilar cholangiocarcinoma: A systematic review and meta-analysis. Medicine.

[4] Wang Y, Ma K, Zhong A, Xiong Q, Chen J (2019). Hepatopulmonary syndrome after radiofrequency ablation of recurrent intrahepatic cholangiocarcinoma: a case report. Onco Targets Ther.

[5] de Rooij T, van Hilst J, van Santvoort H, Boerma D, van den Boezem P, Daams F (2019). Minimally Invasive Versus Open Distal Pancreatectomy (LEOPARD): A Multicenter Patient-blinded Randomized Controlled Trial. Ann Surg.

[6] Wang M, Peng B, Liu J, Yin X, Tan Z, Liu R (2021). Practice Patterns and Perioperative Outcomes of Laparoscopic Pancreaticoduodenectomy in China: A Retrospective Multicenter Analysis of 1029 Patients. Ann Surg.

[7] Jin B, Chen MT, Fei YT, Du SD, Mao YL (2018). Safety and efficacy for laparoscopic versus open hepatectomy: A meta-analysis. Surg Oncol.

[8] Nickel F, Haney CM, Kowalewski KF, Probst P, Limen EF, Kalkum E (2020). Laparoscopic Versus Open Pancreaticoduodenectomy: A Systematic Review and Meta-analysis of Randomized Controlled Trials. Ann Surg.

[9] Hosokawa I, Shimizu H, Yoshidome H, Ohtsuka M, Kato A, Yoshitomi H (2014). Surgical strategy for hilar cholangiocarcinoma of the left-side predominance: current role of left trisectionectomy. Ann Surg.

[10] Gumbs AA, Jarufe N, Gayet B (2013). Minimally invasive approaches to extrapancreatic cholangiocarcinoma. Surg Endosc.

[11] Franken LC, van der Poel MJ, Latenstein AEJ, Zwart MJ, Roos E, Busch OR (2019). Minimally invasive surgery for perihilar cholangiocarcinoma: a systematic review. J Robot Surg.

[12] Hu HJ, Wu ZR, Jin YW, Ma WJ, Yang Q, Wang JK (2019). Minimally invasive surgery for hilar cholangiocarcinoma: state of art and future perspectives. ANZ J Surg.

[13] Agha R, Abdall-Razak A, Crossley E, Dowlut N, Iosifidis C, Mathew G; the STROCSS Group. The STROCSS (2019). Guideline: Strengthening the Reporting of Cohort Studies in Surgery. Int J Surg.

[14] Edge SBBD, Compton CC, Fritz AG, Greene FL, Trotti A. American Joint Committee on cancer. Cancer staging manual: Springer, New York; 2020.

[15] Bismuth H, Nakache R, Diamond T (1992). Management strategies in resection for hilar cholangiocarcinoma. Ann Surg.

[16] Clavien PA, Barkun J, de Oliveira ML, Vauthey JN, Dindo D, Schulick RD (2009). The Clavien-Dindo classification of surgical complications: five-year experience. Ann Surg.

[17] Koch M, Garden OJ, Padbury R, Rahbari NN, Adam R, Capussotti L (2011). Bile leakage after hepatobiliary and pancreatic surgery: a definition and grading of severity by the International Study Group of Liver Surgery. Surgery.

[18] Rahbari NN, Garden OJ, Padbury R, Maddern G, Koch M, Hugh TJ (2011). Post-hepatectomy haemorrhage: a definition and grading by the International Study Group of Liver Surgery (ISGLS). HPB (Oxford).

[19] Rahbari NN, Garden OJ, Padbury R, Brooke-Smith M, Crawford M, Adam R (2011). Posthepatectomy liver failure: a definition and grading by the International Study Group of Liver Surgery (ISGLS). Surgery.

[20] Brookhart MA, Schneeweiss S, Rothman KJ, Glynn RJ, Avorn J, Stürmer T (2006). Variable selection for propensity score models. Am J Epidemiol.

[21] Cipriani F, Ratti F, Fiorentini G, Reineke R, Aldrighetti L (2021). Systematic review of perioperative and oncologic outcomes of minimally-invasive surgery for hilar cholangiocarcinoma. Updates Surg.

[22] Rahnemai-Azar AA, Abbasi A, Tsilimigras DI, Weber SM, Pawlik TM (2020). Current Advances in Minimally Invasive Surgical Management of Perihilar Cholangiocarcinoma. J Gastrointest Surg.

[23] Wang W, Fei Y, Liu J, Yu T, Tang J, Wei F (2021). Laparoscopic surgery and robotic surgery for hilar cholangiocarcinoma: an updated systematic review. ANZ J Surg.

[24] Lin E, Sarmiento JM (2014). Laparoscopic extended right hepatectomy, portal lymphadenectomy, and hepaticojejunostomy for hilar cholangiocarcinoma. J Laparoendosc Adv Surg Tech A.

[25] Yu H, Wu SD, Chen DX, Zhu G (2011). Laparoscopic resection of Bismuth type I and II hilar cholangiocarcinoma: an audit of 14 cases from two institutions. Dig Surg.

[26] Zhang CW, Liu J, Hong DF, Wang ZF, Hu ZM, Huang DS (2018). Pure laparoscopic radical resection for type IIIa hilar cholangiocarcinoma. Surg Endosc.

[27] Yu H, Wu SD, Tian Y, Su Y, Li YN (2013). Single-incision laparoscopic resection of Bismuth I hilar cholangiocarcinoma. Surgical innovation.

[28] Ratti F, Fiorentini G, Cipriani F, Catena M, Paganelli M, Aldrighetti L (2020). Perihilar cholangiocarcinoma: are we ready to step towards minimally invasiveness?. Updates Surg.

[29] Cipriani F, Alzoubi M, Fuks D, Ratti F, Kawai T, Berardi G (2020). Pure laparoscopic versus open hemihepatectomy: a critical assessment and realistic expectations - a propensity score-based analysis of right and left hemihepatectomies from nine European tertiary referral centers. J Hepatobiliary Pancreat Sci.

[30] Cipriani F, Ratti F, Cardella A, Catena M, Paganelli M, Aldrighetti L (2019). Laparoscopic Versus Open Major Hepatectomy: Analysis of Clinical Outcomes and Cost Effectiveness in a High-Volume Center. J Gastrointest Surg.

[31] Li J, Zhao L, Zhang J, Li Z, Li A, Wei Y (2017). Application of the laparoscopic technique in perihilar cholangiocarcinoma surgery. Int J Surg.

[32] Ebata T, Nagino M, Kamiya J, Uesaka K, Nagasaka T, Nimura Y (2003). Hepatectomy with portal vein resection for hilar cholangiocarcinoma: audit of 52 consecutive cases. Ann Surg.

[33] Xu Y, Wang H, Ji W, Tang M, Li H, Leng J (2016). Robotic radical resection for hilar cholangiocarcinoma: perioperative and long-term outcomes of an initial series. Surg Endosc.

[34] Ratti F, Fiorentini G, Cipriani F, Paganelli M, Catena M, Aldrighetti L (2019). Perioperative and Long-Term Outcomes of Laparoscopic Versus Open Lymphadenectomy for Biliary Tumors: A Propensity-Score-Based. Case-Matched Analysis Ann Surg Oncol.

[35] Slim K (2014). Laparoscopy within fast-track or within enhanced recovery after surgery?. Ann Surg.

[36] Ni X, Jia D, Chen Y, Wang L, Suo J (2019). Is the Enhanced Recovery After Surgery (ERAS) Program Effective and Safe in Laparoscopic Colorectal Cancer Surgery? A Meta-Analysis of Randomized Controlled Trials. J Gastrointest Surg.

[37] Morgan KA, Lancaster WP, Walters ML, Owczarski SM, Clark CA, McSwain JR (2016). Enhanced Recovery After Surgery Protocols Are Valuable in Pancreas Surgery Patients. J Am Coll Surg.

[38] Wong-Lun-Hing EM, van Dam RM, van Breukelen GJ, Tanis PJ, Ratti F, van Hillegersberg R (2017). Randomized clinical trial of open versus laparoscopic left lateral hepatic sectionectomy within an enhanced recovery after surgery programme (ORANGE II study). Br J Surg.

[39] Bednarski BK, Nickerson TP, You YN, Messick CA, Speer B, Gottumukkala V (2019). Randomized clinical trial of accelerated enhanced recovery after minimally invasive colorectal cancer surgery (RecoverMI trial). Br J Surg.

[40] Varadhan R, Weiss CO, Segal JB, Wu AW, Scharfstein D, Boyd C (2010). Evaluating health outcomes in the presence of competing risks: a review of statistical methods and clinical applications. Med Care.

[41] Koller MT, Raatz H, Steyerberg EW, Wolbers M (2012). Competing risks and the clinical community: irrelevance or ignorance?. Stat Med.

[42] Willms AG, Schaaf S, Zimmermann N, Schwab R, Güsgen C, Vilz TO (2021). The Significance of Visceral Protection in Preventing Enteroatmospheric Fistulae During Open Abdomen Treatment in Patients With Secondary Peritonitis: A Propensity Score-matched Case-control Analysis. Ann Surg.

[43] Bonnot PE, Piessen G, Kepenekian V, Decullier E, Pocard M, Meunier B (2019). Cytoreductive Surgery With or Without Hyperthermic Intraperitoneal Chemotherapy for Gastric Cancer With Peritoneal Metastases (CYTO-CHIP study): A Propensity Score Analysis. J Clin Oncol.

[44] Jingdong L, Yongfu X, Yang G, Jian X, Xujian H, Jianhua L (2021). Minimally invasive surgery for hilar cholangiocarcinoma: a multicenter retrospective analysis of 158 patients. Surg Endosc.

