# Supplementary material for: Laparoscopic versus open surgery for perihilar cholangiocarcinoma: a multicenter propensity score analysis of short- term outcomes
Source: BMC Cancer. 2023 May 3;23:394. doi: 10.1186/s12885-023-10783-9 (PMC10157952; doi:10.1186/s12885-023-10783-9)
Supplement: Supplementary file 1 — Additional file 1. [file 12885_2023_10783_MOESM1_ESM.docx]

**Contents of supplementary table**

| **Contents** | **Contents** |
| --- | --- |
| Supplementary Table 1 | The detailed Bismuth type of pCCA in each participating centers. |
| Supplementary Table 2 | Baseline characteristics based on Bismuth-Corlett Type before propensity score matching (N=645) |
| Supplementary Table 3 | Intraoperative outcomes based on Bismuth-Corlett Type before propensity score matching. |
| Supplementary Table 4 | Postoperative outcomes based on Bismuth-Corlett Type before propensity score matching. |
| Supplementary Table 5 | Baseline characteristics based on Bismuth-Corlett Type after propensity score matching (N=282) |
| Supplementary Table 6 | Intraoperative outcomes based on Bismuth-Corlett Type after propensity score matching. |
| Supplementary Table 7 | Postoperative outcomes based on Bismuth-Corlett Type before propensity score matching. |
| Supplementary Table 8 | The Fine Gray model analysis of length of stay using raw cohort and matched cohort with considering competing risks. |

**Supplementary Table 1. The detailed Bismuth type of pCCA in each participating centers.**

| **Center** | **Bismuth-Corlett Type** | | | | | | | | | |
| --- | --- | --- | --- | --- | --- | --- | --- | --- | --- | --- |
|  | **I** | | **II** | | **IIIa** | | **IIIb** | | **IV** | |
|  | **LS** | **OP** | **LS** | **OP** | **LS** | **OP** | **LS** | **OP** | **LS** | **OP** |
| Affiliated Hospital of North Sichuan Medical College | 4 | 6 | 4 | 2 | 1 | 2 | 3 | 1 | 3 | 9 |
| The Second Hospital of Hebei Medical University | 23 | 12 | 13 | 12 | 2 | 5 | 9 | 2 | 10 | 3 |
| Hunan Provincial People’s Hospital | 16 | 18 | 28 | 34 | 10 | 3 | 6 | 9 | 11 | 25 |
| Sheng Jing Hospital of China medical university | 1 | 20 | 0 | 10 | 0 | 14 | 1 | 20 | 3 | 35 |
| Ruijin Hospital | 1 | 8 | 3 | 1 | 3 | 0 | 2 | 3 | 6 | 8 |
| Qilu hospital of Shandong university | 17 | 8 | 13 | 6 | 5 | 0 | 4 | 0 | 15 | 0 |
| The Third Affiliated Hospital of Soochow University |  | 3 |  | 4 |  | 1 |  | 1 |  | 6 |
| Tongji Hospital | 2 | 17 | 2 | 8 | 0 | 1 | 2 | 4 | 2 | 5 |
| Affiliated Hospital of Xuzhou Medical University | 3 | 9 | 0 | 5 | 0 | 1 | 1 | 4 | 10 | 2 |
| Zhejiang Provincial People’s Hospital | 5 | 0 | 0 | 0 | 3 | 5 | 4 | 5 | 2 | 4 |
| The First Affiliated Hospital of Chongqing Medical University | 0 | 7 | 0 | 3 | 0 | 3 | 1 | 3 | 2 | 12 |

**Supplemental Table 2. Baseline characteristics based on Bismuth-Corlett Type before propensity score matching (N=645)**

|  | Bismuth-Corlett Type I-II | | | Bismuth-Corlett Type III-IV | | |
| --- | --- | --- | --- | --- | --- | --- |
|  | LS (N=135) | OP (N=193) | P value | LS (N=121) | OP (N=196) | P value |
| Sex, No.(%) |  |  |  |  |  |  |
| Male | 64(47.41) | 126(65.28) | 0.0012 | 65(53.72) | 106(54.08) | 0.9498 |
| Female | 71(52.59) | 67(34.72) |  | 56(46.28) | 90(45.92) |  |
| Age (yrs), mean(SD) | 63.32(10.10) | 62.27(9.15) | 0.3309 | 62.29(9.23) | 61.93(9.22) | 0.7354 |
| BMI (Kg/cm^2^), mean(SD) | 23.33(3.04) | 22.87(2.87) | 0.1601 | 22.89(3.14) | 22.63(2.74) | 0.4413 |
| ASA score, No. (%) |  |  |  |  |  |  |
| I | 24(17.78) | 31(16.06) | 0.8004 | 33(27.27) | 51(26.02) | 0.4829 |
| II | 86(63.70) | 121(62.69) |  | 70(57.85) | 124(63.27) |  |
| III | 25(18.52) | 41(21.24) |  | 18(14.88) | 21(10.71) |  |
| CA199 (U/mL), Median(IQR) | 179.60(50.30~664.20) | 180.90(72.70~372.10) | 0.7991 | 296.00(100.10~843.30) | 348.85(133.25~533.40) | 0.7147 |
| Tbil (μmol/L), Median(IQR) | 47.40(43.70~52.50) | 141.70(72.70~234.30) | <0.0001 | 48.80(44.30~56.40) | 120.90(74.90~216.55) | <0.0001 |
| ALP (μmol/L), Median(IQR) | 346.00(208.00~445.00) | 311.00(223.00~443.00) | 0.1710 | 383.50(226.90~470.00) | 347.65(230.50~489.00) | 0.7347 |
| AST (μmol/L), Median(IQR) | 37.50(32.00~62.40) | 85.00(46.00~147.90) | <0.0001 | 52.00(35.00~112.30) | 92.55(57.25~168.50) | 0.0010 |
| ALT (μmol/L), Median(IQR) | 49.00(23.00~115.60) | 123.00(58.00~189.00) | <0.0001 | 70.80(35.70~151.60) | 108.45(60.55~162.60) | 0.0113 |
| Albumin (μmol/L), Median(IQR) | 35.00(33.00~38.20) | 36.50(33.60~39.60) | 0.0560 | 36.30(34.10~39.00) | 35.65(33.40~38.40) | 0.1116 |
| Maximun Tumor Size(mm), Mean(SD), | 2.80(2.00~3.50) | 2.50(2.00~3.00) | 0.0360 | 3.00(2.50~3.50) | 3.00(2.50~3.50) | 0.9716 |

PSM, Propensity score matching; LS, Laparoscopic surgery; OP, open operation; BMI, body mass index; Tbil, total bilirubin; ALP, A Lkaline Phosphatase; AST, Aspartic acid aminotransferase; ALT, alanine aminotransferase.

*Fisher exact test.

**Supplemental Table 3. Intraoperative outcomes based on Bismuth-Corlett Type before propensity score matching.**

| Demographics | Bismuth-Corlett Type I-II | | | Bismuth-Corlett Type III-IV | | |
| --- | --- | --- | --- | --- | --- | --- |
|  | LS (N=135) | OP (N=193) | P value | LS (N=121) | OP (N=196) | P value |
| Conversion to laparotomy, No. (%) | 7(5.19) | 0(0.00) | 0.0014 | 12(9.92) | 0(0.00) | <0.0001 |
| Hepatectomy, No. (%) |  |  |  |  |  |  |
| Bile duct only | 55(40.74) | 130(67.36) | 0.0001 | 38(31.40) | 43(21.94) | 0.0064 |
| Left hemihepatectomy | 62(45.93) | 45(23.32) |  | 60(49.59) | 98(50.00) |  |
| Right hemihepatectomy | 9(6.67) | 12(6.22) |  | 10(8.26) | 45(22.96) |  |
| Left Segmentectomy | 1(0.74) | 0(0.00) |  | 3(2.48) | 3(1.53) |  |
| Right Segmentectomy | 1(0.74) | 0(0.00) |  | 5(4.13) | 2(1.02) |  |
| Bile duct and part of hepatectomy | 7(5.19) | 6(3.11) |  | 5(4.13) | 5(2.55) |  |
| Operative time (min), mean(SD) | 330.00(253.00~420.00) | 307.00(253.00~376.00) | 0.5556 | 360.00(300.00~425.00) | 360.00(319.00~400.00) | 0.1028 |
| No. lymph node, mean(SD) | 9.00(7.00~12.00) | 10.00(8.00~12.00) | 0.0663 | 10.00(9.00~12.00) | 12.00(9.00~14.00) | 0.0628 |
| Vascular resection, No. (%) | 10(7.41) | 25(12.95) | 0.1094 | 12(9.92) | 66(33.67) | <.0001 |
| Biliary reconstruction, N (%) |  |  | 0.0014 |  |  | <.0001 |
| Choledochojejunostomy | 88(71.54) | 97(53.30) |  | 42(39.25) | 29(15.85) |  |
| Hepaticojejunostomy | 35(28.46) | 85(46.70) |  | 65(60.75) | 154(84.15) |  |
| Biliary plasty, No. (%) | 26(19.26) | 82(42.49) | <0.0001 | 48(39.67) | 108(55.10) | 0.0076 |
| Caudate lobectomy, No. (%) | 17(12.69) | 24(12.50) | 0.9601 | 121(100.00) | 196(100.00) | - |
| IBL(ml), mean(SD) | 200.00(100.00~400.00) | 200.00(100.00~400.00) | 0.7645 | 350.00(200.00~600.00) | 400.00(200.00~600.00) | 0.4310 |
| Transfusion during surgery, No.(%) | 33(24.44) | 69(35.75) | 0.0295 | 47(38.84) | 81(41.33) | 0.6615 |
| R0, No. (%) | 2(1.48) | 5(2.59) | 0.4939 | 18(14.88) | 25(12.76) | 0.5921 |

PSM, Propensity score matching; LS, Laparoscopic surgery; OP, open operation; IBL, Intraoperative blood loss.

*Fisher exact test.

**Supplemental Table 4. Postoperative outcomes based on Bismuth-Corlett Type before propensity score matching.**

| **Demographics** | **Bismuth-Corlett Type I-II** | | | **Bismuth-Corlett Type III-IV** | | |
| --- | --- | --- | --- | --- | --- | --- |
|  | **LS (N=135)** | **OP (N=193)** | **P value** | **LS (N=121)** | **OP (N=196)** | **P value** |
| Major complications, No. (%) | 23(17.04) | 50(25.91) | 0.0574 | 39(32.23) | 86(43.88) | 0.0393 |
| Hemorrhage | 3(2.22) | 8(4.15) | 0.3411 | 14(11.57) | 11(5.61) | 0.0559 |
| Biliary fistula | 7(5.19) | 7(3.63) | 0.4921 | 12(9.92) | 22(11.22) | 0.7148 |
| Abdominal abscess | 8(5.93) | 18(9.33) | 0.2619 | 12(9.92) | 29(14.80) | 0.2086 |
| Hepatic insufficiency | 0(0.00) | 1(0.52) | 0.4022 | 3(2.48) | 8(4.08) | 0.4489 |
| Gastrointestinal fistula | 1(0.74) | 2(1.04) | 0.7820 | 0(0.00) | 2(1.02) | 0.2650 |
| Incision infection | 3(2.22) | 7(3.63) | 0.4665 | 1(0.83) | 9(4.59) | 0.0624 |
| Pneumonia | 6(4.44) | 7(3.63) | 0.7088 | 10(8.26) | 21(10.71) | 0.4756 |
| Renal failure | 1(0.74) | 3(1.55) | 0.5088 | 2(1.65) | 7(3.57) | 0.3177 |
| Heart failure | 1(0.74) | 4(2.07) | 0.3326 | 1(0.83) | 10(5.10) | 0.0433 |
| ARDS | 1(0.74) | 3(1.55) | 0.5088 | 2(1.65) | 6(3.06) | 0.4374 |
| CD stage ≥ III, No. (%) | 18(13.33) | 38(19.69) | 0.1322 | 13(10.74) | 51(26.02) | 0.0010 |
| Reoperation, No. (%) | 2(1.48) | 5(2.59) | 0.4939 | 3(2.48) | 2(1.02) | 0.3112 |
| Death (30d), No. (%) | 2(1.48) | 8(4.15) | 0.1673 | 6(4.96) | 16(8.16) | 0.2754 |
| Death (90d), No. (%) | 4(2.96) | 9(4.66) | 0.4373 | 8(6.61) | 18(9.18) | 0.4175 |
| Time of off-bed activity(d), median(IQR) | 4.00(3.00~6.00) | 5.00(4.00~6.00) | 0.1725 | 4.00(3.00~7.00) | 5.00(3.00~7.00) | 0.7983 |
| PDTK(d), median(IQR) | 7.00(4.00~10.00) | 9.00(6.00~14.00) | 0.0011 | 8.00(5.00~12.00) | 8.00(5.00~15.50) | 0.0454 |
| LOS (d), Median(IQR) | 12.00(9.00~16.00) | 14.00(12.00~19.00) | 0.0006 | 14.00(10.00~20.00) | 17.00(13.00~23.50) | 0.0021 |

PSM, Propensity score matching; LS, Laparoscopic surgery; OP, open operation; ARDS, Acute Respiratory Distress Syndrome; CD, Clavien-Dindo; PDTK, postoperative drainage tube keep time; LOS, length of stay.

*Fisher exact test.

**Supplemental Table 5.** **Baseline characteristics based on Bismuth-Corlett Type after propensity score matching (N=282)**

| **Demographics** | **Bismuth-Corlett Type I-II** | | | **Bismuth-Corlett Type III-IV** | |  |
| --- | --- | --- | --- | --- | --- | --- |
|  | **LS (N=68)** | **OP (N=62)** | **P value** | **LS (N=73)** | **OP (N=79)** | **P value** |
| Sex, No.(%) |  |  |  |  |  |  |
| Male | 38(55.88) | 40(64.52) | 0.3156 | 48(65.75) | 46(58.23) | 0.3411 |
| Female | 30(44.12) | 22(35.48) |  | 25(34.25) | 33(41.77) |  |
| Age (yrs), mean(SD) | 63.44(9.15) | 64.24(8.63) | 0.6095 | 61.73(9.14) | 62.18(9.28) | 0.7634 |
| BMI (Kg/cm^2^), mean(SD) | 22.69(20.31~23.88) | 22.43(20.96~23.71) | 0.9473 | 22.48(3.15) | 22.45(2.44) | 0.9473 |
| ASA score, No. (%) |  |  |  |  |  |  |
| I | 16(23.53) | 9(14.52) | 0.3744 | 23(31.51) | 25(31.65) | 0.6483 |
| II | 39(57.35) | 42(67.74) |  | 38(52.05) | 45(56.96) |  |
| III | 13(19.12) | 11(17.74) |  | 12(16.44) | 9(11.39) |  |
| CA199 (U/mL), Median(IQR) | 195.25(82.55~406.15) | 150.00(64.60~281.30) | 0.0076 | 256.00(131.40~612.00) | 288.00(101.90~456.30) | 0.5457 |
| Tbil (μmol/L), Median(IQR) | 48.50(44.15~54.30) | 46.80(20.90~86.80) | 0.9181 | 50.60(44.80~87.10) | 74.50(42.10~119.20) | 0.8085 |
| ALP (μmol/L), Median(IQR) | 363.45(209.00~440.00) | 244.00(189.30~403.00) | 0.4003 | 323.30(216.80~440.00) | 320.00(186.00~450.00) | 0.5882 |
| AST (μmol/L), Median(IQR) | 44.95(34.25~98.90) | 67.65(34.20~119.00) | 0.7380 | 78.00(44.80~146.00) | 78.00(54.30~122.90) | 0.6471 |
| ALT (μmol/L), Median(IQR) | 60.00(34.95~121.90) | 108.15(53.30~138.00) | 0.4224 | 105.70(51.80~179.40) | 87.20(56.70~154.00) | 0.2631 |
| Albumin (μmol/L), Median(IQR) | 34.90(32.15~38.10) | 35.85(32.30~39.70) | 0.2662 | 36.20(34.10~38.50) | 35.60(32.50~38.30) | 0.2460 |
| Maximun Tumor Size(mm), Mean(SD), | 2.50(2.00~3.00) | 2.50(2.00~3.00) | 0.6082 | 2.80(2.30~3.50) | 3.00(2.50~3.50) | 0.2067 |

PSM, Propensity score matching; LS, Laparoscopic surgery; OP, open operation; BMI, body mass index; Tbil, total bilirubin; ALP, A Lkaline Phosphatase; AST, Aspartic acid aminotransferase; ALT, alanine aminotransferase.

*Fisher exact test.

**Supplemental Table 6. Intraoperative outcomes based on Bismuth-Corlett Type after propensity score matching.**

| **Demographics** | **Bismuth-Corlett Type I-II** | | | **Bismuth-Corlett Type III-IV** | | |
| --- | --- | --- | --- | --- | --- | --- |
|  | **LS (N=68)** | **OP (N=62)** | **P value** | **LS (N=73)** | **OP (N=79)** | **P value** |
| Conversion to laparotomy, No. (%) | 3(4.41) | 0(0.00) | 0.0943 | 8(10.96) | 0(0.00) | 0.0025 |
| Hepatectomy, No. (%) |  |  |  |  |  |  |
| Bile duct only | 42(61.76) | 41(66.13) | 0.7622 | 24(32.88) | 16(20.25) | 0.0512 |
| Left hemihepatectomy | 15(22.06) | 12(19.35) |  | 32(43.84) | 40(50.63) |  |
| Right hemihepatectomy | 5(7.35) | 6(9.68) |  | 7(9.59) | 19(24.05) |  |
| Left Segmentectomy | 0(0.00) | 0(0.00) |  | 2(2.74) | 1(1.27) |  |
| Right Segmentectomy | 0(0.00) | 0(0.00) |  | 5(6.85) | 1(1.27) |  |
| Bile duct and part of hepatectomy | 6(8.82) | 3(4.84) |  | 3(4.11) | 2(2.53) |  |
| Operative time (min), mean(SD) | 330.00(260.00~420.00) | 300.00(260.00~366.00) | 0.1907 | 380.00(320.00~420.00) | 360.00(328.00~400.00) | 0.3445 |
| No. lymph node, mean(SD) | 9.00(7.50~11.50) | 10.00(8.00~12.00) | 0.4559 | 12.00(9.00~13.00) | 12.00(9.00~14.00) | 0.7389 |
| Vascular resection, No. (%) | 7(10.29) | 9(14.52) | 0.4643 | 8(10.96) | 22(27.85) | 0.0090 |
| Biliary reconstruction, N (%) |  |  | 0.0925 |  |  | 0.0006 |
| Choledochojejunostomy | 43(69.35) | 31(54.39) |  | 25(37.31) | 9(12.33) |  |
| Hepaticojejunostomy | 19(30.65) | 26(45.61) |  | 42(62.69) | 64(87.67) |  |
| Biliary plasty, No. (%) | 20(29.41) | 25(40.32) | 0.1915 | 41(56.16) | 49(62.03) | 0.4626 |
| Caudate lobectomy, No. (%) | 12(17.91) | 3(4.92) | 0.0225 | 73(100.00) | 79(100.00) |  |
| IBL(ml), mean(SD) | 200.00(100.00~500.00) | 200.00(100.00~400.00) | 0.4570 | 400.00(200.00~600.00) | 400.00(200.00~600.00) | 0.6639 |
| Transfusion during surgery, No.(%) | 19(27.94) | 21(33.87) | 0.4644 | 28(38.36) | 31(39.24) | 0.9110 |
| R0, No. (%) | 55(80.88) | 52(83.87) | 0.6556 | 60(82.19) | 67(84.81) | 0.6635 |

PSM, Propensity score matching; LS, Laparoscopic surgery; OP, open operation; IBL, Intraoperative blood loss.

*Fisher exact test.

**Supplemental Table 7. Postoperative outcomes based on Bismuth-Corlett Type before propensity score matching.**

| **Demographics** | **Bismuth-Corlett Type I-II** | | | **Bismuth-Corlett Type III-IV** | | |
| --- | --- | --- | --- | --- | --- | --- |
|  | **LS (N=68)** | **OP (N=62)** | **P value** | **LS (N=73)** | **OP (N=79)** | **P value** |
| Major complications, No. (%) | 19(27.94) | 15(24.19) | 0.6272 | 25(34.25) | 30(37.97) | 0.6327 |
| Hemorrhage | 3(4.41) | 2(3.23) | 0.7254 | 7(9.59) | 3(3.80) | 0.1502 |
| Biliary fistula | 7(10.29) | 0(0.00) | 0.0094 | 7(9.59) | 7(8.86) | 0.8767 |
| Abdominal abscess | 7(10.29) | 6(9.68) | 0.9068 | 10(13.70) | 11(13.92) | 0.9679 |
| Hepatic insufficiency | 0(0.00) | 0(0.00) |  | 2(2.74) | 4(5.06) | 0.4623 |
| Gastrointestinal fistula | 1(1.47) | 2(3.23) | 0.5056 | 0(0.00) | 2(2.53) | 0.1712 |
| Incision infection | 3(4.41) | 4(6.45) | 0.6068 | 0(0.00) | 4(5.06) | 0.0514 |
| Pneumonia | 4(5.88) | 5(8.06) | 0.6245 | 8(10.96) | 4(5.06) | 0.1781 |
| Renal failure | 1(1.47) | 2(3.23) | 0.5056 | 2(2.74) | 3(3.80) | 0.7149 |
| Heart failure | 1(1.47) | 1(1.61) | 0.9475 | 1(1.37) | 2(2.53) | 0.6069 |
| ARDS | 1(1.47) | 2(3.23) | 0.5056 | 2(2.74) | 1(1.27) | 0.5140 |
| CD stage ≥ III, No. (%) | 16(23.53) | 14(22.58) | 0.8980 | 10(13.70) | 18(22.78) | 0.1488 |
| Reoperation, No. (%) | 2(2.94) | 2(3.23) | 0.9252 | 2(2.74) | 2(2.53) | 0.9362 |
| Death (30d), No. (%) | 2(2.94) | 5(8.06) | 0.1961 | 5(6.85) | 7(8.86) | 0.6459 |
| Death (90d), No. (%) | 2(2.94) | 6(9.68) | 0.1104 | 7(9.59) | 6(7.59) | 0.6605 |
| Time of off-bed activity(d), median(IQR) | 5.00(3.00~6.00) | 4.50(3.00~6.00) | 0.3549 | 4.00(3.00~7.00) | 4.00(3.00~7.00) | 0.1318 |
| PDTK(d), median(IQR) | 9.00(6.00~12.50) | 10.00(6.00~13.00) | 0.8474 | 8.00(5.00~12.00) | 8.00(5.00~12.00) | 0.1446 |
| LOS (d), Median(IQR) | 13.00(10.50~19.00) | 14.00(12.00~20.00) | 0.0474 | 14.00(12.00~19.00) | 16.00(13.00~23.00) | 0.0074 |

PSM, Propensity score matching; LS, Laparoscopic surgery; OP, open operation; ARDS, Acute Respiratory Distress Syndrome; CD, Clavien-Dindo; PDTK, postoperative drainage tube keep time; LOS, length of stay.

*Fisher exact test.

**Supplemental Table 8. The Fine Gray model analysis of length of stay using raw cohort and matched cohort with considering competing risks.**

| Risk Factors | Matched Cohort | | | |  | Matched Cohort | | | |
| --- | --- | --- | --- | --- | --- | --- | --- | --- | --- |
|  | Univariate analysis | | Multivariate analysis | |  | Univariate analysis | | Multivariate analysis | |
|  | HR(95%CI) | P value | HR(95%CI) | P value |  | HR(95%CI) | P value | HR(95%CI) | P value |
| **Surgery** |  |  |  |  |  |  |  |  |  |
| LS | Reference |  | Reference |  |  | Reference |  | Reference |  |
| OP | 0.67(0.56,0.81) | <.0001 | 0.77(0.63~0.95) | 0.013 |  | 0.69(0.55,0.88) | 0.002 | 0.73(0.58~0.92) | 0.008 |
| Female | 1.30(1.08,1.57) | 0.005 |  |  |  | 0.95(0.74,1.21) | 0.656 |  |  |
| Age ≥ 60y | 1.07(0.88,1.31) | 0.497 |  |  |  | 1.03(0.81,1.30) | 0.835 |  |  |
| BMI, Kg/cm^2^ |  |  |  |  |  |  |  |  |  |
| <18.5 | Reference |  |  |  |  | Reference |  |  |  |
| 18.5~24 | 1.30(0.79,2.15) | 0.308 |  |  |  | 0.72(0.42,1.23) | 0.225 |  |  |
| >24 | 1.21(0.99,1.48) | 0.069 |  |  |  | 1.13(0.82,1.54) | 0.462 |  |  |
| ASA |  |  |  |  |  |  |  |  |  |
| I | Reference |  |  |  |  | Reference |  |  |  |
| II | 0.86(0.71,1.06) | 0.151 |  |  |  | 0.88(0.69,1.12) | 0.297 | 0.86(0.67~1.11) | 0.254 |
| III | 0.79(0.60,1.05) | 0.107 |  |  |  | 0.59(0.39,0.88) | 0.010 | 0.57(0.38~0.85) | 0.006 |
| Tumor size > 3cm | 1.31(1.08,1.59) | 0.008 |  |  |  | 0.98(0.75,1.28) | 0.883 |  |  |
| Lymphnodes > 8 | 0.68(0.55,0.84) | 0.001 |  |  |  | 0.96(0.73,1.26) | 0.778 |  |  |
| Preoperative Tbil >85.5μmol/L | 0.77(0.64,0.92) | 0.005 |  |  |  | 0.74(0.56,0.97) | 0.027 | 0.75(0.57~0.98) | 0.038 |
| Preoperative ALP ≥110μmol/L | 0.54(0.34,0.87) | 0.011 |  |  |  | 1.03(0.45,2.39) | 0.941 |  |  |
| Preoperative AST >40μmol/L | 0.56(0.46,0.70) | <.0001 |  |  |  | 0.76(0.56,1.03) | 0.074 |  |  |
| Preoperative ALT >40μmol/L | 0.51(0.41,0.64) | <.0001 | 0.60(0.48~0.76) | <.0001 |  | 0.71(0.51,0.99) | 0.046 |  |  |
| CA199, U/ml |  |  |  |  |  |  |  |  |  |
| ≤ 50 | Reference |  |  |  |  | Reference |  |  |  |
| 50~400 | 0.67(0.51,0.88) | 0.004 |  |  |  | 0.69(0.49,0.97) | 0.032 |  |  |
| ≥ 400 | 0.71(0.53,0.95) | 0.022 |  |  |  | 0.67(0.46,0.98) | 0.039 |  |  |
| Operating time, min |  |  |  |  |  |  |  |  |  |
| ≤ 200 | Reference |  |  |  |  | Reference |  |  |  |
| 200~400 | 0.84(0.61,1.15) | 0.270 |  |  |  | 1.08(0.66,1.78) | 0.751 |  |  |
| ≥ 400 | 0.62(0.44.89) | 0.008 |  |  |  | 1.17(0.71,1.95) | 0.539 |  |  |
| Intraoperative blood loss, ml |  |  |  |  |  |  |  |  |  |
| ≤ 100 | Reference |  |  |  |  | Reference |  | Reference |  |
| 100~500 | 0.61(0.50,0.76) | <.0001 | 0.61(0.49~0.77) | <.0001 |  | 0.82(0.62,1.09) | 0.166 | 0.86(0.64~1.16) | 0.326 |
| ≥ 500 | 0.42(0.33,0.54) | <.0001 | 0.42(0.32~0.55) | <.0001 |  | 0.65(0.49,0.87) | 0.003 | 0.68(0.50~0.92) | 0.011 |
| Transfusion during surgery | 0.57(0.47,0.70) | <.0001 |  |  |  | 0.68(0.53,0.87) | 0.002 |  |  |
| Hepatectomy |  |  |  |  |  |  |  |  |  |
| Bile duct only | Reference |  |  |  |  | Reference |  |  |  |
| Left hemihepatectomy | 1.21(0.99,1.48) | 0.068 | 1.54(1.23~1.92) | <.0001 |  | 1.06(0.80,1.40) | 0.685 |  |  |
| Right hemihepatectomy | 0.50(0.36,0.69) | <.0001 | 0.74(0.52~1.04) | 0.087 |  | 0.80(0.59,1.09) | 0.164 |  |  |
| Left Segmentectomy | 0.31(0.08,1.23) | 0.095 | 0.45(0.11~1.75) | 0.248 |  | 1.04(0.40,2.67) | 0.943 |  |  |
| Right Segmentectomy | 0.82(0.38,1.79) | 0.617 | 0.93(0.40~2.14) | 0.855 |  | 0.70(0.34,1.48) | 0.354 |  |  |
| Bile duct and part of hepatectomy | 0.84(0.52,1.36) | 0.475 | 0.79(0.50~1.25) | 0.318 |  | 0.96(0.54,1.71) | 0.881 |  |  |
| Conversion to laparotomy | 0.73(0.39,1.38) | 0.330 |  |  |  | 0.67(0.38,1.19) | 0.169 |  |  |
| Vascular resection |  |  |  |  |  |  |  |  |  |
| None | Reference |  |  |  |  | Reference |  |  |  |
| Hepatic artery | 0.73(0.49,1.09) | 0.120 |  |  |  | 0.77(0.53,1.13) | 0.177 |  |  |
| Portal vein | 0.53(0.32,0.87) | 0.012 |  |  |  | 0.66(0.46,0.94) | 0.020 |  |  |
| Hepatic artery & Portal vein | 0.34(0.22,0.53) | <.0001 |  |  |  | 0.57(0.36,0.89) | 0.014 |  |  |
| Vascular reconstruction | 0.47(0.28,0.77) | 0.003 |  |  |  | 0.68(0.53,0.88) | 0.003 |  |  |
| Digestive reconstruction |  |  |  |  |  |  |  |  |  |
| Choledochojejunostomy | Reference |  |  |  |  | Reference |  |  |  |
| Hepaticojejunostomy | 0.57(0.47,0.70) | <.0001 |  |  |  | 0.76(0.60,0.98) | 0.034 |  |  |
| Biliary plasty | 0.75(0.63,0.91) | 0.003 |  |  |  | 1.01(0.80,1.27) | 0.965 |  |  |
| TNM |  |  |  |  |  |  |  |  |  |
| I(T1N0M0) | Reference |  |  |  |  | Reference |  |  |  |
| II(T2a/2bN0M0) | 1.02(0.77,1.35) | 0.905 |  |  |  | 0.90(0.62,1.32) | 0.592 |  |  |
| IIIA(T3N0M0) | 0.86(0.59,1.24) | 0.411 |  |  |  | 0.98(0.59,1.62) | 0.935 |  |  |
| IIIB(T4N0M0) | 1.48(0.95,2.32) | 0.083 |  |  |  | 1.01(0.54,1.90) | 0.976 |  |  |
| IVA(T, N2M0) | 0.85(0.62,1.17) | 0.319 |  |  |  | 0.80(0.53,1.22) | 0.299 |  |  |
| IVB(T,N,M1) | 0.94(0.59,1.51) | 0.800 |  |  |  | 0.89(0.49,1.61) | 0.706 |  |  |
